# Bilateral Word Selectivity Gradients in the Visual Word Form System in Skilled Deaf Readers

**DOI:** 10.1162/NOL.a.13

**Published:** 2025-09-17

**Authors:** Laurie S. Glezer, Stephen McCullough, Brennan Terhune-Cotter, Karen Emmorey

**Affiliations:** San Diego State University Research Foundation, San Diego, CA, USA; Joint Doctoral Program in Language and Communicative Disorders, San Diego State University, San Diego, CA, USA; and University of California, San Diego, San Diego, CA, USA; School of Speech, Language and Hearing Sciences, San Diego State University, San Diego, CA, USA

**Keywords:** deaf readers, fMRI, ventral occipitotemporal cortex, visual word form system

## Abstract

In hearing people visual word recognition relies on a hierarchical organization in left ventral occipitotemporal (vOT) cortex. While right hemisphere recruitment has been implicated in poor reading, this may not be the case for deaf readers as there is evidence that for skilled deaf readers the right vOT is also engaged during word recognition. However, the nature of representations along the vOT hierarchy and the degree of laterality in skilled deaf readers remain largely unknown. This study aimed to examine the hierarchical organization for written words in the vOT bilaterally for skill-matched deaf and hearing readers to determine whether deafness and phonological ability modulates the laterality of word-selectivity gradients. Using fMRI, we employed the same design as previous studies, presenting stimuli that represent a scale of orthographic regularity: consonant strings, pseudowords, and real words. For hearing readers, our results replicate previous findings showing a hierarchical structure solely in the left visual word form system (VWFS). For deaf readers, we find this same hierarchical structure in the left VWFS, but we also observe a similar hierarchical structure in the right VWFS. Unlike studies that show maladaptive right hemisphere activation in people with dyslexia, the bilateral tuning to written words seen in our study is not maladaptive since all participants were skilled readers. The bilateral hierarchical organization of the VWFS represents a unique neural signature for successful reading in deaf adults and suggests that the typical developmental shift from bilateral to predominantly left-lateralized processing is not necessary for successful reading.

## INTRODUCTION

Reading is a complex cognitive skill that develops over time, is experience dependent, and relies on a distributed neural circuitry associated with different component processes. Over the past three decades, understanding the neural underpinnings of reading in hearing children and adults has received considerable attention, leading to an ever clearer understanding of the neural circuitry involved in reading. Visual word recognition relies on a hierarchy of increasingly larger and more abstract neural representations that lay along a posterior-to-anterior gradient in left ventral occipitotemporal (vOT) cortex, collectively referred to as the *visual word form system* (VWFS; Dehaene et al., 2005; Olulade et al., 2013; Vinckier et al., 2007). More specifically, it is posited that the left ventral visual stream contains neurons that are tuned to increasingly abstract features, running from posterior to anterior regions. Posterior regions are tuned to simple features like lines and oriented bars, while more anterior regions are tuned to more complex representations, like whole letters and letter combinations and in the most anterior region, neurons are tuned to whole words. Functional magnetic resonance imaging (fMRI) studies in hearing readers reveal a gradient pattern of activation along the left ventral stream that correlates with the complexity of the visual stimuli (Olulade et al., 2013, 2015; Vinckier et al., 2007). The hierarchical nature and selectivity of this region in hearing skilled readers appears to be specific to the left hemisphere, as the homologous region in the right hemisphere does not show a hierarchical structure or selectivity for written words (Glezer et al., 2015; Olulade et al., 2013, 2015; Vinckier et al., 2007).

This hierarchical organization and specialization for written words in the VWFS is experience dependent and develops with reading acquisition (Brem et al., 2010; Dehaene et al., 2005; Glezer et al., 2009; Olulade et al., 2013; Szwed et al., 2014; Vinckier et al., 2007; Zhao et al., 2014). Several studies have demonstrated that as children learn to read, activation in the left VWFS increases while activation in the right VWFS decreases, such that as reading proficiency increases activation shifts from primarily bilateral to predominantly left-lateralized (Chyl et al., 2018; Olulade et al., 2013; Wandell & Yeatman, 2013). This pattern suggests a critical role for the left occipitotemporal cortex in skilled reading. Olulade et al. (2013) examined the hierarchical structure in the VWFS in children and adults and found that children (mean age = 10 yr) exhibited more bilateral activation during word processing, while adults showed left-lateralized activation, particularly in the anterior occipitotemporal cortex. In adults, word-selective activity occurred in more anterior and lateral regions compared to children, who showed stronger activation in more posterior regions. This posterior to anterior shift reflects a developmental progression where word processing becomes more efficient and localized to the left hemisphere as reading skills mature.

Studies examining people with reading difficulties also show altered activation patterns when compared to skilled reading. When reading single words, people with dyslexia show reduced activation in the left fusiform gyrus, but increased activation in the right fusiform gyrus (Kronschnabel et al., 2013; Maisog et al., 2008; Pugh et al., 2001). This bilateral pattern of activation is thought to be compensatory but inefficient, as indicated by the persistent reading difficulties characteristic of dyslexia. Studies that have examined the hierarchical structure within the VWFS in people with dyslexia show an absence of the typical word selectivity gradients seen in skilled readers (Olulade et al., 2015; van der Mark et al., 2009). In people with dyslexia, the left hemisphere gradient is absent with no significant differences observed in word selectivity between posterior and anterior regions. This disruption of typical word tuning development in the left hemisphere may result in the continued involvement of the right hemisphere in people with dyslexia as a compensatory, albeit inefficient, mechanism for visual word recognition.

Electrophysiological studies using event-related potentials (ERPs) have also shed light on the neural processes that underlie visual word recognition. In particular, the N170 ERP component, a negative going wave peaking around 170 ms after word onset, is thought to index visual word form processing. This component is robust in skilled readers when they read written words (Amora et al., 2022), and skilled hearing readers often exhibit a more pronounced left-lateralized N170 response to words compared to nonwords or other stimuli (e.g., Maurer, Brandeis, & McCandliss, 2005). The amplitude, latency, and laterality of the N170 is modulated by reading skill (Maurer, Brem, et al., 2005). For example, in individuals with reading difficulties, the N170 is often less robust, with differences in amplitude, latency, and laterality compared to skilled readers. Additionally, as reading develops, the N170 appears to become more sensitive to word forms, as indicated by an increasing amplitude, faster latency, and lateralization shifting to the left (Fraga González et al., 2014). Thus, ERP and fMRI studies report similar findings regarding left hemisphere dominant processing in skilled hearing readers, with right hemisphere involvement observed only during development or with those who have reading difficulties.

An influential hypothesis which aims to explain the left laterality seen with skilled hearing reading and the differing response patterns associated with reading development and reading skill is the phonological mapping hypothesis (PMH; McCandliss & Noble, 2003). The PMH posits that the ability to process and accurately link graphemes to phonemes is essential for skilled reading to develop. Furthermore, the PMH posits that the emergence of left lateralized processing for visual word forms is due to the need to link the visual orthographic word forms with left hemisphere auditory language regions. This then explains, for the hearing population, the difference in laterality in people with developing or disordered phonological processing. Sacchi and Laszlo (2016) argued that an important prediction of the PMH is that better phonological awareness should result in a more left lateralized neural response. This study examined this prediction in 5th and 6th grade children while they viewed words. Consistent with the PMH, they reported that the degree of left lateralization of the N170 was predicted by phonological awareness (but not by vocabulary size).

While the PMH might hold for hearing people, it is less clear whether the PMH holds true for deaf people or if it can account for reading success in deaf people for whom phonology appears to be less strongly involved in reading. Although there is some evidence linking phonological skill with reading ability in deaf children (see Mayer & Trezek, 2014, for a review), studies with deaf adults have shown that (a) phonological awareness is not as clearly linked to reading success as for hearing adults (e.g., Mayberry et al., 2011; Sehyr & Emmorey, 2022), (b) phonological knowledge is relatively coarse-grained for deaf readers (e.g., Hirshorn et al., 2015; McQuarrie & Parrila, 2009), and (c) phonological codes may not be automatically accessed during word reading for either deaf adults (e.g., Bélanger et al., 2013; Costello et al., 2021) or deaf children (Ormel et al., 2010). Nonetheless, it has recently been proposed that similar to hearing readers, less skilled deaf readers may engage phonology to a greater extent than skilled deaf readers (Holcomb et al., 2024; but see Bélanger et al., 2012).

The neural architecture supporting reading in deaf people is less well understood, but recent studies have shed some light on the neural underpinnings of reading in deaf adults (see Emmorey & Lee, 2021, for a review). Results generally show similar left hemisphere VWFS activation and specificity between deaf and hearing readers (Aparicio et al., 2007; Emmorey et al., 2013; Glezer et al., 2018; Wang et al., 2015) including evidence that skilled deaf readers, like skilled hearing readers, exhibit tuning to whole words in a subsection of the left VWFS, termed the *visual word form area* (VWFA; Glezer et al., 2018). This finding indicates that the nature of orthographic tuning in the VWFA is not altered by imprecise phonological representations as the deaf readers in this study had relatively poor phonological abilities. Similarly, Wang et al. (2015) found that the location, activation strength, and connectivity pattern of the left VWFA were comparable for deaf and hearing readers, despite the poorer speech skills of the deaf participants in this study. These findings suggest that the specificity of the left VWFA can develop without robust phonological tuning or access to speech sounds.

Interestingly, both Wang et al. (2015) and Glezer et al. (2018) found that deaf readers also appeared to engage the right VWFA, which is typically not reported for hearing readers. Consistent with these fMRI results, ERP studies have shown that the N170 component, which may be generated by the VWFS (Brem et al., 2009; Hirshorn et al., 2016; Nobre et al., 1994), exhibits more bilateral activation in deaf readers compared to hearing readers (Emmorey et al., 2017; Sehyr et al., 2020). Furthermore, a higher amplitude of the N170 in the right hemisphere was associated with better reading and spelling abilities in deaf readers. In contrast, hearing readers with poorer reading skills showed larger N170 amplitudes in the right hemisphere (Emmorey et al., 2017). Thus, although recruitment of the right hemisphere has been implicated in poor reading for hearing people (Shaywitz et al., 2002; Shaywitz & Shaywitz, 2005), this does not appear to be the case for skilled deaf readers, indicating that neural adaptations that are maladaptive for hearing readers may actually be beneficial for deaf readers. This pattern of results points to a potential unique neural signature for skilled reading in deaf individuals: engagement of both the left and right VWFS in single word reading. However, the exact nature of the neural representation and degree of laterality and tuning for word and word-like stimuli along the entirety of the bilateral VWFS for skilled reading in deaf people has yet to be fully examined.

As noted above, both deaf and hearing skilled readers strongly engage the left VWFS and exhibit a finely tuned representation for written words in a subsection of the VWFS (i.e., the VWFA—a smaller region within the VWFS), thus establishing a clear role for the left VWFS in successful reading. However, both fMRI and ERP research suggest that the role of the right hemisphere differs between deaf and hearing skilled readers. In the case of hearing readers with developing or poor phonological abilities, engagement of the right hemisphere appears to be either due to ongoing development or is maladaptive. While in deaf skilled readers, whose phonological representations are less precise, right hemisphere involvement appears to be effective and beneficial (Emmorey et al., 2017; Sehyr et al., 2020). However, the exact nature of the representations in the entire VWFS (not just the VWFA) in both the left and right hemispheres has never been examined in deaf skilled readers. While phonological skill may be at the heart of the left hemisphere specialization for written words for hearing readers as posited by the PMH, it is also possible that some other factor that drives both reading skill and phonological awareness is behind the laterality seen in readers with better phonological awareness. Examining the neural architecture along the VWFS in deaf readers can help address this question. If skilled deaf readers exhibit a left-lateralized hierarchical representation for word and word-like stimuli, then phonological mapping from letters to sounds cannot be the sole driving force behind the left lateralization seen in skilled reading. However, if there is a right-lateralized or bilateral hierarchical representation for deaf readers, it would lend support to the PMH account of lateralization in hearing readers.

To examine these questions, we employed the same design used in other studies that have examined the laterality and hierarchical representation for written words within the ventral visual stream in hearing individuals (Olulade et al., 2013, 2015; Vinckier et al., 2007). We used stimuli that represent a scale of orthographic regularity—consonant strings, pseudowords, and real words, along with a low-level task that imposes a constant strategic processing load across all stimuli (Wilson et al., 2013) and is not confounded by linguistic factors or skill level. We present results examining the hierarchical organization and hemispheric lateralization in the ventral visual stream, in both skilled deaf and hearing readers.

## MATERIALS AND METHODS

The current study is part of a larger project examining the neural architecture underlying phonological and orthographic processing in deaf and hearing readers. In this article, we present the methods and data from the conditions that specifically addressed the current research questions.

### Participants

Fifteen deaf and fourteen hearing right-handed adults participated in the study. One hearing subject was unable to complete the scanning session after experiencing peripheral nerve stimulation. One deaf subject was unable to stay alert and awake for the entirety of the session. These two subjects were excluded from the results reported in this article. Therefore, the deaf group comprised 14 deaf adults (6 women, mean age 34 [±5.5]), and the hearing group comprised 13 hearing adults (8 women, mean age 31 [±7.4]). All participants reported no history of a learning disability or a neurological or behavioral disorder. The deaf participants were prelingually deaf (i.e., before age 2), had a hearing loss of 70 dB or greater (unaided), acquired American Sign Language (ASL) prior to age 5, and used ASL as a primary language. All participants had received a high school diploma or higher education (deaf = 17.1 yr of education [±1.8] and hearing = 15.5 yr of education [±2.2]). The hearing readers were all monolingual in English, while the deaf readers were bilingual in ASL and English. We elected to compare the deaf readers with monolingual English speakers because both groups read in only one language (there is no written form of ASL) and because it is not clear what group of bilinguals constitutes the most appropriate comparison group (i.e., it is unclear what first or second language is parallel to ASL).

Informed consent was obtained from all participants according to procedures approved by the San Diego State University (SDSU) Human Research Protection Program.

Table 1 details the demographic data and the average scores of the participants in this study. We matched participant groups on reading level using the reading comprehension subtest of the Peabody Individual Achievement Test–Revised (PIAT; Markwardt, 1998). In this subtest, participants silently read a sentence, then choose a picture from among four choices that best matches the sentence. Items increase in difficulty throughout the test, and the test is discontinued if a participant produces five errors among seven consecutive responses. The mean reading score for the deaf group was 90 (±5.8, range 79–99), and for the hearing group the mean reading score was 92 (±6.0, range 76–98); this difference was not statistically significant (*p* = 0.314). On average, both the deaf and hearing groups were considered skilled readers according to PIAT scoring measures. Importantly, the participant groups did differ in phonological skills, which were measured using phonological awareness tests developed by Hirshorn et al. (2015). These phonological skill tests were specifically designed for profoundly deaf adults and do not require overt speech production. The tests examine the extent to which participants base phonological decisions on orthography and their ability to access phonological representations without useful orthographic cues. The mean phonological score for the deaf group was 65% (±19%) and for the hearing group the mean phonological score was 95% (±7%); this difference was statistically significant (*p* = 0.00006, *t* test).

**Table 1.** Assessment measure means for the deaf and hearing participant groups

	Deaf	Hearing
	Mean (*SD*)	Mean (*SD*)
	*N* = 14	*N* = 13
Age	34 (5.5)	31 (7.4)
Gender	6 women	8 women
Years of education	17.1	15.5
PIAT raw score	90 (5.8)	92 (6.0)
Reading age (yr)	16 (2.0)	17 (1.7)
Reading grade	11th	12th
English phonological task (percent correct)	65 (19)	95 (7)

*Note*. Standard deviations are given in parentheses. PIAT = Peabody Individual Achievement Test–Revised.

### Stimuli, Design, and Procedure

This study included three conditions of interest and a fixation baseline condition (there was an additional condition associated with the larger project that was not included in this analysis). Because we were interested in examining activation related to processing written stimuli that have an increasing orthographic similarity to real words, we included three conditions: consonant strings (CS), pseudowords (PW), and real words (RW) similar to Vinckier et al. (2007). The studies by Olulade et al. (2013, 2015) used only real words and false fonts. All stimuli were 3–6 letters long, and stimulus groups were matched on average length (average length = 5 letters). RW stimuli were generated and measured using the English Lexicon Project (Balota et al., 2007) and were all monomorphemic, high-frequency nouns and verbs (average Log_Freq_HAL = 11.2, range = 8.5–15.4, bigram frequency = 1,756) according to the Hyperspace Analogue to Language (HAL) frequency norms (Lund & Burgess, 1996), The PW were all pronounceable and monomorphemic nonwords (e.g., no “s” on the end) and were generated using the ARC wordform database to contain only orthographically existing onsets and bodies. The CS were generated using a random letter sequence generator (Reed, 2002) to include only consonants. These stimuli were checked to ensure none were pronounceable or contained orthographically legal letter combinations or existing onsets and bodies (Rastle et al., 2002).

All participants completed three fMRI runs, and each run consisted of eight written stimuli blocks with 60 stimuli in each block presented for 100 ms with a 200 ms interstimulus interval. Each written stimuli block lasted 18 s. These blocks were separated by the baseline fixation blocks (18 s). Conditions were presented twice within a run (randomly presented) for a total of two blocks per condition per run. There was a total of 360 written stimuli per condition. Each run lasted approximately 5 min, for a total of 15 min scanning time.

#### Task

Participants were asked to press a button when the written stimuli changed color. To ensure that the participant had to pay attention and could not predict when to press the button; between two and six stimuli were colored and randomly presented in each block.

### Data Acquisition

MRI data were collected at the SDSU Imaging Center with a Siemens Magnetom Prisma 3 Tesla MRI scanner equipped with a 30-channel head coil. The functional images were acquired over three runs. Forty-five interleaved slices in a transverse orientation (3 mm thick, no gap) were acquired using an echo-planar imaging (EPI) sequence (flip angle = 60°, TR = 1 s, TE = 31 ms, FOV = 216°). High-resolution T1-weighted anatomical images were also acquired for each participant. The stimuli were projected onto a screen attached to the bore opening at the foot of the scanner using a MacBook Pro computer running PsyScope X Build 77 (Bonatti, n.d.). The participants viewed the stimuli via a mirror positioned atop the head coil.

### Data Preprocessing

Preprocessing and most statistical analyses were done using the SPM12 software package (Ashburner et al., 2021). After discarding the first five volumes of each run, EPI images were spatially realigned, resliced to 2 × 2 × 2 mm^3^, and spatially normalized to a standard MNI reference brain. Images were then smoothed with an isotropic 8 mm Gaussian kernel. After removing low-frequency temporal noise from the EPI runs with a high-pass filter (1/128 Hz), fMRI responses were modeled with a design matrix comprising the onset of trial types and movement parameters as regressors of interest and of no interest, respectively, using a standard canonical hemodynamic response function (HRF).

### Data Analysis

#### Ventral visual word form system identification

**Figure 1.**
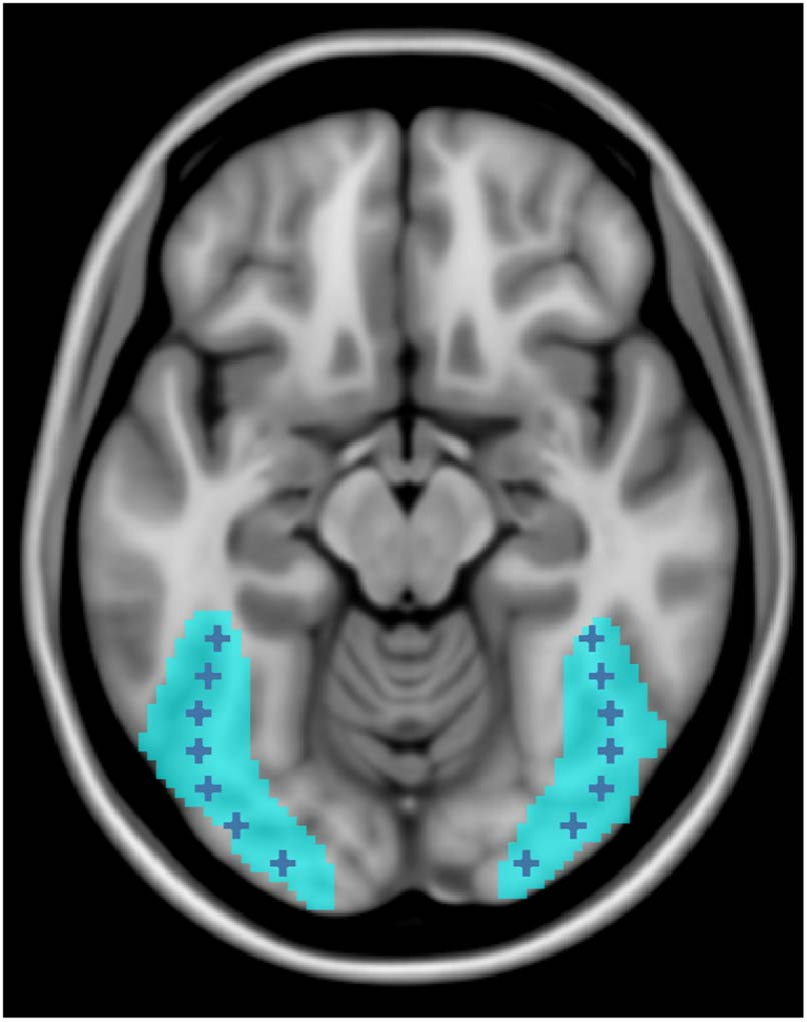
Region of interest (ROI) locations within the study defined activation region (SDAR). Activation from both groups to identify the left and right SDARs are shown in cyan. ROI locations within the SDAR (cyan) are denoted by dark blue crosses.

To focus our analysis on the occipitotemporal region in both the left and right hemispheres that was reliably active in our dataset, while also ensuring we were examining valid and unbiased regions in both participant groups, we conducted a whole-brain analysis to isolate these regions (similar to Olulade et al., 2013, 2015, and Vinckier et al., 2007). First, we conducted a first-level analysis for the experimental stimuli (RW, PW, and CS) versus fixation (Fix) contrast and obtained activation maps for each individual participant. We then generated a group activation map by using a one-sample *t* test at the second level that included all participants (*p* < 0.001, corrected). Within the left hemisphere and right hemisphere separately, we selected the activation cluster that was located within and spanned the fusiform gyrus and was in a similar location to previous studies (Olulade et al., 2013, 2015; Vinckier et al., 2007). We did this for both the left and the right hemispheres (left cluster size = 3,073 voxels, right cluster size = 2,664 voxels). These study defined activation regions (SDARs; see Figure 1) were then used to focus our analyses, as detailed below.

#### Gradient mapping images

To examine the hierarchy for word-selectivity in the vOT cortex in both the deaf and hearing groups, we analyzed the data in a manner similar to Vinckier et al. (2007), who also examined activation related to stimuli with increasing orthographic similarity to RW. First, to normalize for differences in activation, we created ratio images for each participant by dividing the contrast of PW versus Fix and CS versus Fix by the contrast of RW versus Fix. Then within-group (deaf and hearing) activation maps were created using the resulting ratio images—(PW vs. Fix)/(RW vs. Fix) or (CS vs. Fix)/(RW vs. Fix)—for each participant in a one-sample *t* test. To focus results on the occipitotemporal cortex, we inclusively masked the within-group results with the SDAR (the regions identified in the whole-brain analysis as outlined above). The resulting group ratio images for (PW vs. Fix)/(RW vs. Fix) and (CS vs. Fix)/(RW vs. Fix) indexes the degree to which (0%–100%) each condition of interest (i.e., PW and CS) activates in comparison to RW. That is, areas that show activation of 100% are activated by the stimuli of interest as much as RW and conversely, regions showing very little, or no activation compared to RW will be closer to 0%.

#### Region of interest analysis

To evaluate the statistical significance of the gradient mapping results, we conducted a region of interest (ROI) analysis. We examined ROIs by defining coordinates along the posterior-anterior hierarchy in the vOT region. The ROIs were built using similar methods reported in Olulade et al. (2013, 2015) and Vinckier et al. (2007). The coordinates used in these studies served as a guide for this study. First, we identified, along the *y* axis, the most posterior and anterior activation points of the SDAR. Then using Marsbar (Brett et al., 2002), we plotted seven spherical, nonoverlapping ROIs (4 mm radius) every 8 mm along the posterior-anterior gradient (i.e., the *y* axis) in the left hemisphere. We then adjusted the *x* and *z* coordinates to ensure the entirety of each ROI fell within the SDAR, thus ensuring we were sampling from areas that reliably exhibited activation associated with single word reading. Next, we created seven homotopic spheres at the same locations in the right hemisphere. The locations of the ROIs were as follows: Montreal Neurological Institute (MNI) coordinates: (±25 −92 −10), (±36 −84 −12), (±42 −76 −14), (±45 −68 −14), (±44 −60 −16), (±42 −52 −16), (±40 −44 −14). Figure 1 shows where the ROIs fall within the SDAR. Finally, using Marsbar (Brett et al., 2002) we extracted the percent signal change in each ROI for each condition in each participant.

We then conducted a series of statistical analyses. First, we conducted a repeated measures analysis of variance (ANOVA) separately for the deaf and hearing groups using ROI, Condition, and Hemisphere as within-subject factors. We then followed this with planned paired *t* tests (*p* = 0.05, two-tailed) on the percent signal change comparing each condition. Finally, due to the complex structure of the group data, we conducted a follow-up generalized linear mixed-effects model (GLMM) to examine the interactions of the within- and between-subject variables, and included the factors of Group, Hemisphere, ROI, and Condition, accounting for both fixed and random effects.

## RESULTS

We first present results from the gradient images which allows us to visualize the hierarchy for word-selectivity in the vOT cortex. The gradient maps are ratio images for (PW vs. Fix)/(RW vs. Fix) and (CS vs. Fix)/(RW vs. Fix), which indexes the degree to which each condition of interest (i.e., PW and CS) activates in comparison to RW. These maps are nonstatistical representations of the hierarchy. To determine the statistical significance of the hierarchy, we conducted a series of statistical analyses. First, we report the results of the repeated measures ANOVA separately for the deaf and hearing groups using ROI, Condition, and Hemisphere as within-subject factors. We then present the results of the planned paired *t* tests (*p* = 0.05, two-tailed) on percent signal change comparing each condition. Finally, we report the follow-up generalized GLMM analysis, which examined the interactions of the within and between subject variables and included the factors of Group, Hemisphere, ROI, and Condition, accounting for both fixed and random effects.

### Gradient Images

**Figure 2.**
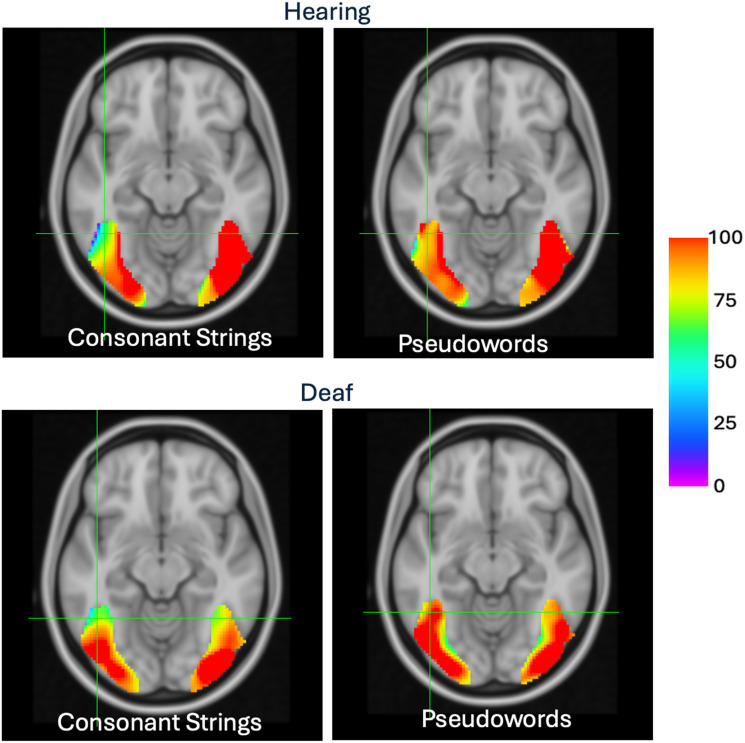
Gradient images demonstrating the spatial layout of sensitivity to visual word forms. Top row shows sensitivity maps for hearing readers and bottom row shows sensitivity maps for deaf readers. The crosshair position is located in the region of the putative visual word form area. The color bar indicates the percent activation of consonant strings (leftmost panels) and pseudowords (rightmost panels) relative to real words.

For the hearing readers, we observed the expected hierarchical pattern of responses to word and word-like stimuli in the left hemisphere along the ventral axis (Figure 2, top row). As demonstrated in previous studies, when compared to RW, PW appear to activate similarly for almost the entire VWFS region, whereas CS appear to gradually decrease in activation moving posterior to anterior, demonstrating the pattern that is expected for neuronal sensitivity to word and word-like stimuli. In addition, this pattern is evident only in the left hemisphere. The right hemisphere shows no such gradient, with activation that appears nearly equivalent for PW and CS. Our results replicate previous findings for typical adult readers (Olulade et al., 2013, 2015; Vinckier et al., 2007).

For the deaf readers we also find a hierarchical pattern in the left hemisphere for words and word-like stimuli, paralleling the results for hearing readers. This finding suggests that the left hemisphere is tuned to the visual word form similarly in both deaf and hearing readers. However, in contrast to the hearing group, the right hemisphere in the deaf group also showed a pattern that would suggest tuning to word and word-like stimuli, which was almost identical to that found in the left hemisphere.

### Region of Interest Analysis

**Figure 3.**
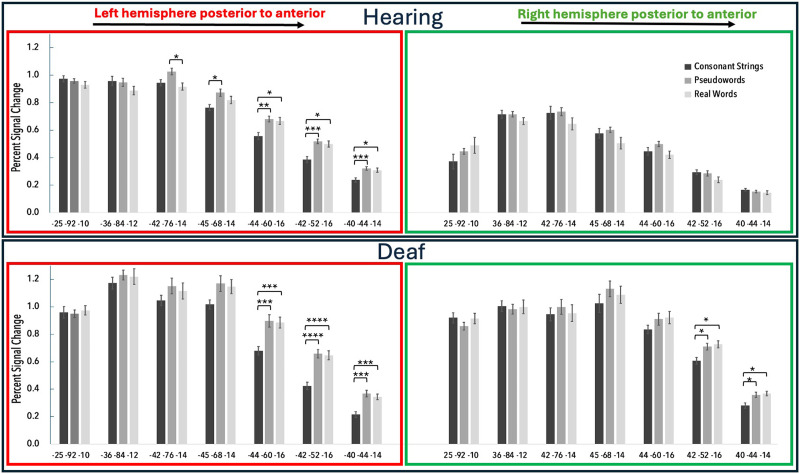
Regions of interest analysis. Panels show the percent signal change in each ROI for hearing readers (top panel) and deaf readers (bottom panel) in the left (red) and right (green) hemispheres. Error bars represent within-subject standard error of the mean. *p < 0.05, **p < 0.01, ***p < 0.005, ****p < 0.001.

To determine if the gradient image results are statistically significant, we conducted an ROI analysis. Our analysis on the percent signal change within each plotted ROI (Figure 3) reveals that in the hearing group, the left hemisphere (top panel, red outline) shows the expected pattern of increasing selectivity as you move anteriorly for word and word-like stimuli, but this gradient is not apparent in the right hemisphere (top panel, green outline). A repeated measures ANOVA with ROI, Condition, and Hemisphere as within-subject factors, revealed a significant main effect of ROI, *F*(6, 72) = 11.06, *p* = 0.0015, and Hemisphere, *F*(1, 12) = 8.97, *p* = 0.011. Additionally, significant interactions were found for ROI × Hemisphere, *F*(6, 72) = 21.40, *p* = 0.0003, and Condition × ROI × Hemisphere, *F*(12, 144) = 3.15, *p* = 0.034. Additionally, there was a trend for a significant interaction between Condition × ROI, *F*(12, 144) = 2.70, *p* = 0.065. Planned *t* tests revealed that the three most anterior ROI in the left hemisphere: (−44 −60 −16), (−42 −52 −16), and (−40 −44 −14) show a significant difference between RW and CS (*p* = 0.032, 0.013, and 0.025, paired *t* tests, respectively) and PW and CS (*p* = 0.008, 0.002, and 0.004, paired *t* tests, respectively). However, in the right hemisphere, there were no significant differences between any of the conditions in any of the ROIs for the hearing readers.

In the left hemisphere, deaf readers showed a very similar response profile to the hearing readers, demonstrating gradient sensitivity to written words, with only the three most anterior ROIs: (−44 −60 −16), (−42 −52 −16), and (−40 −44 −14) showing a significant difference between RW and CS (*p* = 0.002, 0.001, and 0.002, paired *t* tests, respectively) and PW and CS (*p* = 0.004, 0.0002, and 0.003, paired *t* tests, respectively). However, the deaf group also showed this same selectivity to word and word-like stimuli in the right hemisphere in the two most anterior ROIs: (42 −52 −16) and (40 −44 −14) with a significant difference between RW and CS (*p* = 0.012 and 0.012, paired *t* tests, respectively) and PW and CS (*p* = 0.027 and 0.038, paired *t* tests, respectively). A repeated measures ANOVA revealed a significant main effect of ROI, *F*(6, 78) = 4.159, *p* = 0.011, but not Hemisphere, *F*(1, 13) = 0.317, *p* = 0.54. Significant two-way interactions were found for Condition × ROI, *F*(12, 156) = 5.909, *p* = 0.0014, and ROI × Hemisphere, *F*(6, 78) = *p* < 0.0001, and a three-way interaction was found for Condition, ROI, and Hemisphere, *F*(12, 156) = 6.444, *p* = 0.0007.

To further examine if the results in the right hemisphere were significantly different between the deaf and hearing groups, we conducted a GLMM using Hemisphere, ROI, Condition, and Groups as factors. Results from the GLMM show main effects of ROI (Estimate = 0.474, *p* = 0.005), Condition (Estimate = 1.124, *p* = 0.0018), and Hemisphere (Estimate = 1.203, *p* = 0.0118). Significant two-way interactions were observed between Group and ROI (Estimate = −0.272, *p* = 0.0102), Group and Condition (Estimate = −0.469, *p* = 0.0321), and Hemisphere and ROI (Estimate = −0.318, *p* = 0.0029). Additionally, three-way interactions between Group, ROI, and Hemisphere (Estimate = 0.177, *p* = 0.0082), and Condition, ROI, and Hemisphere (Estimate = 0.139, *p* = 0.0049) were significant. The four-way interaction between Group, ROI, Condition, and Hemisphere was also significant (Estimate = −0.072, *p* = 0.0195). Overall, these results show that while the left hemisphere in the deaf group responded similarly to that of the hearing group, only the deaf group showed a hierarchical profile in the right hemisphere with hierarchical selectivity to word and word-like stimuli that was similar to the left hemisphere.

## DISCUSSION

This study presents novel evidence indicating that skilled deaf readers exhibit a distinct neural signature in the ventral visual stream, differentiating them from their hearing counterparts. We replicated previous findings in hearing readers, demonstrating the well-established left-lateralized hierarchical tuning to visual word forms. We show deaf readers also exhibit a hierarchical organization within the left hemisphere’s ventral visual stream, characterized by a posterior-to-anterior gradient of sensitivity to visual word forms. However, a key difference emerged: unlike hearing readers—who typically show minimal right hemisphere engagement for word or word-like stimuli—deaf readers displayed a hierarchical response profile in the right hemisphere, mirroring the left hemisphere’s selectivity.

In hearing readers, the right hemisphere’s involvement in reading is generally only evident in less proficient readers, either due to developmental factors or dyslexia. This right hemisphere engagement in hearing readers is often seen as compensatory or maladaptive, reflecting challenges in phonological processing or reading development because there is not a hierarchical response profile that reflects specific tuning to words or word-like stimuli. In contrast, our findings of bilateral tuning to written words in deaf readers shows that right hemisphere engagement is present; further, the activation pattern demonstrates the hierarchical organization thought to be crucial for successful reading to occur (Olulade et al., 2013, 2015). This finding challenges the notion that right hemisphere involvement is necessarily maladaptive, indicating instead that it may play a critical role in successful reading in deaf individuals. However, while it is tempting to conclude that the hierarchical organization observed in the right hemisphere is functional and effective because all participants in the study were proficient readers, further studies that include less-skilled readers are needed to confirm this. Additionally, further studies that include more variability in reading, hearing levels, and phonological abilities are needed to determine if the bilateral activation pattern we see represents an effective adaptive neural response to reduced auditory input and reduced reliance on phonological processing.

The PMH provides a framework for understanding the left hemisphere dominance observed in skilled hearing readers, positing that this lateralization arises from the need to map sounds to letters—a process that requires the integration of visual word forms with the left hemisphere’s speech regions. The developmental trajectory of reading skills highlights the role of the right hemisphere in reading. During the early stages of reading acquisition, children exhibit more bilateral activation, with both hemispheres participating in the processing of visual word forms (Brem et al., 2010; Dehaene et al., 2005; Olulade et al., 2013; Vinckier et al., 2007). As children learn to read, and with the maturation of phonological processing skills, neural engagement typically shifts from bilateral to predominantly left-lateralized activation, reflecting increasing proficiency in mapping visual representations of words to their auditory phonological counterparts (Chyl et al., 2018; Olulade et al., 2013; Wandell & Yeatman, 2013). For hearing readers, the right hemisphere’s role diminishes as the left hemisphere becomes more specialized in processing written language. This left-lateralized activation is thought to be essential for efficient reading, as it allows for the rapid and automatic recognition of words.

However, this left hemisphere specialization does not occur uniformly across all hearing readers. In individuals with dyslexia, or those with underdeveloped phonological processing, the right hemisphere may remain engaged during reading tasks (Kronschnabel et al., 2013; Maisog et al., 2008; Pugh et al., 2001). This right hemispheric activation in dyslexic readers is often interpreted as a compensatory mechanism, possibly indicating a reliance on visual and/or orthographic strategies rather than phonological processing or reflecting an attempt to recruit additional neural resources to overcome difficulties in phonological processing. Yet, because reading difficulties continue, the recruitment of the right hemisphere for dyslexic readers suggests that the compensatory right hemispheric involvement may not be sufficient to support skilled reading in this population and is therefore maladaptive. However, Olulade et al. (2015) demonstrate that people with dyslexia do not exhibit any hierarchical organization when reading written words in either the left or the right hemisphere. This finding suggests that there is a failure to establish hierarchical processing for reading in its entirety and the lack of the development of specific hierarchical representations for written words is either “a cause or a consequence” (Olulade et al., 2015, p. 750) of their reading difficulties. It is possible that dyslexic readers recruit the right hemisphere for visual word processing, which may contribute to the slower, error prone, and more effortful reading that characterizes this population. However, for deaf readers, because they demonstrate hierarchical representations in both the left and right VWFS, it suggests that the right hemisphere activation represents the establishment of a distinct and successful reading circuit.

Several studies have revealed that the right vOT appears to be recruited and modifiable especially under atypical developmental conditions (e.g., blindness), after intensive visual training, or following lesions to the left vOT. Blind individuals exhibit cross-modal plasticity in the right vOT, thought to reflect the recruitment of this area for tactile or auditory processing (see Saccone et al., 2024, for a review). These findings suggest a potential for the right vOT to adapt to novel sensory experiences. Additionally, intensive visual training with novel scripts or stimuli (e.g., Gauthier et al., 2000; Mei et al., 2013) or acquiring expertise in visual categories such as radiologic images or musical notation (Harley et al., 2009; Proverbio et al., 2013, 2024) results in enhanced right vOT responses, indicating functional plasticity driven by explicit training or experience. We note that the experience of bilingualism does not alter the left-laterality of the VWFA, at least not for same-script bilinguals (Boukrina et al., 2014). Finally, patients who present with left-hemisphere damage involving the VWFS region often show compensatory recruitment of homologous right-hemisphere regions, indicating that the right vOT is capable of substantial functional adaptation (e.g., Cohen et al., 2004). Very interestingly, and highly relevant for our study, recent research with (hearing) individuals who have right lateralized language following a large left hemisphere perinatal stroke involving language regions, but crucially not the VWFA, demonstrated visual word form processing lateralized to the right hemisphere where language was located, providing further strong support for the PMH (Seydell-Greenwald et al., 2023).

While the PMH effectively explains reading success in hearing individuals, it does not account for the proficiency achieved by deaf readers, for whom phonology plays a less central role (Mayberry et al., 2011; Sehyr & Emmorey, 2022). Deaf readers do not depend as heavily on phonological awareness for reading success. Their phonological representations tend to be less precise (due to an absence of acoustic features), and they may not automatically engage phonological codes during word reading (Bélanger et al., 2013; Costello et al., 2021). Therefore, examining the VWFS in deaf readers offers a unique opportunity to test whether phonological mapping is the sole driver of left lateralization in reading. Our findings of tuning to word and word-like stimuli in both the left and right VWFS in deaf individuals do support the PMH, suggesting that left lateralized language regions involved in phonological mapping from letters to sounds is a significant factor in establishing the left lateralization for visual word form processing observed in skilled reading among hearing individuals. However, for deaf people, because phonological skills do not play as large a role in learning to read, or in being a skilled reader, these abilities therefore do not play a central role in hemispheric mapping, and thus activation may remain bilateral. Nonetheless, deaf readers may rely to some extent on phonological information while learning to read until direct orthographic-to-semantic connections can be established. Until more studies are conducted to examine the neural tuning, hierarchical structure, and laterality for people with less-skilled reading, both deaf and hearing, our conclusions regarding the role of phonology in lateralization remain tentative—a limitation of our study.

Our study raises the question of whether phonological mapping is the only route to skilled reading. The finding that deaf readers exhibit tuning to word and word-like stimuli in both the left and right VWFS challenges the existing belief that sound-to-letter mapping is fundamental to developing skilled reading. Instead, these results suggest that while left-lateralized regions may be essential for phoneme-to-grapheme mapping in hearing readers, additional mechanisms must account for the bilateral activation observed in deaf skilled readers. In the absence of a finely tuned phonological component, the neural architecture of deaf readers appears to adapt, developing a hierarchical structure tuned to written words in both hemispheres. This adaptation may be driven by several factors, including the influence of sign language and fingerspelling, changes in visual processing associated with deafness, differences in orthographic and/or phonological knowledge, or reduced reliance on spoken phonology. While our study was not designed to include these factors (and this is a limitation of the study), we consider these ideas in the following sections.

One possible (but speculative) factor that may influence the mapping and organization of the VWFS in deaf readers is the acquisition of fingerspelling during childhood. Fingerspelling is a representation of the orthographic system of a spoken language in which each letter corresponds to distinct hand configurations. Fingerspelled words are an integral component of the ASL lexicon, are frequently used, and are learned in early childhood—even before learning to read (Brentari & Padden, 2001; see Lee & Secora, 2022, for review). Fingerspelling may recruit both bilateral and right-lateralized body-part recognition regions within the fusiform gyrus (Peelen & Downing, 2005; Taylor et al., 2007). Some evidence for this hypothesis comes from neuroimaging studies showing that recognition of fingerspelled words activates the fusiform gyrus bilaterally (Emmorey et al., 2015; Waters et al., 2007). Moreover, there is a growing body of evidence indicating that fingerspelling skills are closely linked with reading proficiency in deaf individuals such that good fingerspellers tend to be good readers (Emmorey & Petrich, 2012; Sehyr & Emmorey, 2022; Stone et al., 2015). Fingerspelling is also used as an instructional technique in deaf education to support the recognition and memory for printed words, thus amplifying the association between fingerspelled and printed words (Haptonstall-Nykaza & Schick, 2007; Humphries & MacDougall, 1999). These findings taken together raise a question that could be explored in future studies: Does the right hemisphere’s involvement in processing fingerspelled letters carry over to reading printed words, thus contributing to the bilateral activation patterns observed in skilled deaf readers? This idea is similar to the PMH in hearing readers, which promotes left hemisphere lateralization, but in this case, fingerspelling plays a role in maintaining and/or refining the responsivity of both the left and right ventral visual pathways for processing word forms. Further studies are necessary to directly test this relationship by investigating the neural correlates of fingerspelling and reading in deaf individuals, as well as exploring how fingerspelling experience and/or skill influences the development of the reading network. Gaining a deeper understanding of the specific mechanisms through which fingerspelling impacts reading could provide valuable insights into the neural basis of literacy in deaf people.

Another factor that we speculate may influence the laterality of the neural representations for visual word forms in deaf readers is their enhanced perceptual span during reading. The perceptual span is the region around fixation where useful visual information can be obtained when reading. For hearing readers, this region extends 3–4 characters to the left of fixation and 14–15 characters to the right of fixation (Rayner, 1998). In contrast, deaf readers have been shown to have a leftward span of 10 characters (Stringer et al., 2024) and a rightward span of 18 characters (Bélanger et al., 2012). Larger left and right perceptual spans for deaf readers may be a result of visual attentional changes associated with deafness (e.g., Proksch & Bavelier, 2002) and/or sign language use (e.g., Stoll & Dye, 2019). An interesting question is whether this larger leftward span could lead to more text being processed in the left visual hemifield, thus increasing right hemisphere engagement as compared to hearing readers. Further research is required to more fully understand the implications of an expanded perceptual span on the neural organization for visual word processing in deaf individuals.

One factor not yet examined, but that would be predicted by the PMH in relation to our findings, is that spoken language phonology does not play the same integral role in reading development for deaf individuals as it does for hearing individuals. As a result, phonology may never “come online” or be integrated into the reading process in the same way it is for hearing people. Unlike people with dyslexia, who need to rely on phonology to learn to read but lack the necessary phonological skills, deaf individuals may bypass phonological processing during reading acquisition. Instead of integrating imprecise spoken phonological information and recruiting the right hemisphere as a compensatory mechanism, as is often seen in dyslexia, deaf readers might beneficially engage the right hemisphere using other modalities or inputs, such as visual or orthographic processing, sign language, or fingerspelling. This alternative recruitment could promote tuning in both hemispheres for reading. A key prediction, then, would be that less skilled deaf readers may show diminished or altered tuning in the right hemisphere, or potentially bilaterally. Some evidence for this prediction is that poorer deaf readers (Emmorey et al., 2017) and poorer deaf spellers (Sehyr et al., 2020) show a smaller right hemisphere N170 response. Investigating the neural correlates in deaf readers could provide valuable insights into the role of different linguistic inputs in reading development and further refine our understanding of the neural mechanisms and linguistic components necessary for successful reading.

Because our findings demonstrate a potentially important role for the right hemisphere in skilled reading, it is important to also point out that the right hemisphere VWFS has been shown to be modulated by other factors such as script and task. To date, the majority of studies examining the neural representation in the VWFS have been conducted with alphabetic scripts. However, several studies have suggested that the right hemisphere appears to play a larger role in logographic scripts, like Chinese, than what is typically seen in alphabetic scripts (see Guo et al., 2022, for a review). Chinese character recognition tends to elicit activity in the bilateral VWFS with some research showing stronger activity in the right VWFS. The right hemisphere contribution during Chinese reading is thought to be the result of the requirement for more holistic processing strategies needed during early visual and orthographic analyses. However, this research is far from conclusive, and the specific roles and the extent of right hemisphere involvement in Chinese reading continue to be areas of active research. Further, it appears that task effects follow the same pattern. For alphabetic scripts, task can alter the amount of activation in the left VWFS such that a phonological task will increase activation when compared to a perceptual task (Mano et al., 2013). For logographic scripts, this same task effect is present in both the left and right VWFS (Qu et al., 2022).

In conclusion, our study provides compelling evidence that the neural mechanisms underlying reading can adapt flexibly depending on the sensory and linguistic experiences of the reader. The bilateral neural tuning in skilled deaf readers underscores the brain’s ability to develop alternative pathways for achieving reading proficiency, independent of phonological processing. These findings have important implications for theories of reading development, suggesting that multiple neural routes can support skilled reading, and highlight the need to consider diverse populations in reading research to fully understand the brain’s capacity for adaptation.

## ACKNOWLEDGMENTS

We would like to thank Lucinda O’Grady Farnady for her help recruiting participants. We also thank all of our participants without whom this research would not be possible.

## FUNDING INFORMATION

Karen Emmorey, National Science Foundation (https://dx.doi.org/10.13039/100000001), Award ID: BCS-1756403. Karen Emmorey, National Institute on Deafness and Other Communication Disorders (https://dx.doi.org/10.13039/100000055), Award ID: R01 DC010997.

## AUTHOR CONTRIBUTIONS

**Laurie S. Glezer**: Conceptualization; Data curation; Formal analysis; Investigation; Methodology; Project administration; Visualization; Writing – original draft; Writing – review & editing. **Stephen McCullough**: Conceptualization; Investigation; Methodology; Writing – review & editing. **Brennan Terhune-Cotter**: Investigation; Methodology; Writing – review & editing. **Karen Emmorey**: Conceptualization; Funding acquisition; Methodology; Writing – review & editing.

## DATA AVAILABILITY STATEMENT

Stimuli, masks, ROIs and fMRI data analysis code are available at the Open Science Framework (https://osf.io/627kd/?view_only=8cc89db2cc9941eab1db6c82d48ff461). Further details may be requested from the authors.

## TECHNICAL TERMS

Visual word recognition: The cognitive process of rapidly and accurately identifying written words by integrating visual input with stored linguistic knowledge.
Visual word form system (VWFS): An area within the vOT specialized for processing written language, transforming visual information into abstract orthographic representations for reading. The VWFS has a hierarchical organization and extends from the initial stages of visual processing in primary visual cortex to the anterior fusiform gyrus.
Hierarchical organization: The structured processing of sensory information through successive stages. For vision, this process starts in primary visual cortex, where initially basic perceptual features are processed (e.g., oriented bars). As you progress anteriorly in the ventral visual pathway, increasingly complex, abstract representations are formed (e.g., words) in higher order cortical regions (e.g., anterior fusiform gyrus).
Bilateral: Processing certain cognitive tasks or sensory inputs relatively equally in both the left and right hemispheres.
Fusiform gyrus: A structure on the ventral surface of the temporal and occipital lobes. It is implicated in high-level visual object recognition including (but not limited to) written words. Other terms often used include ventral visual pathway and occipitotemporal gyrus.
Laterality: The dominance or specialization of one hemisphere over the other in processing certain cognitive tasks or sensory inputs, such as language.
Orthographic regularity: The consistency with which written language maps letters or letter combinations (orthography) onto their corresponding spoken sounds (phonology). A language with high orthographic regularity (e.g., Italian, Spanish) has consistent spelling-to-sound mappings and thus it is easier to predict a word’s pronunciation from its spelling. A language with low orthographic regularity (e.g., English, French) has inconsistent spelling-to-sound mappings, which makes it difficult to predict a word’s pronunciation based purely on its spelling.
Visual word form area (VWFA): A small region within the left VWFS that specializes in processing whole written words. Damage to this area results in a specific deficit in being able to visually recognize written words, called pure alexia.
